# The segmented flavivirus ALSV-encoded nucleoprotein VP2 inhibits type I interferon production by targeting RIG-I

**DOI:** 10.1128/spectrum.02484-25

**Published:** 2026-01-28

**Authors:** Mingming Pan, Zhixia Song, Mengmeng Wang, Mengru Zhao, Shu Fang, Fangyu Jin, Qianqian Tan, Wenbo Xu, Lihe Che, Nan Liu, Liyan Sui, Quan Liu, Zhijun Hou, Yinghua Zhao

**Affiliations:** 1 College of Wildlife and Protected Area, Northeast Forestry University 673337 , Harbin, China; 2 Department of Infectious Diseases and Center of Infectious Diseases and Pathogen Biology, Key Laboratory of Organ Regeneration and Transplantation of the Ministry of Education, State Key Laboratory for Diagnosis and Treatment of Severe Zoonotic Infectious Diseases, Key Laboratory for Zoonosis of the Ministry of Education, The First Hospital of Jilin University 117971, Changchun, China; Regional Centre for Biotechnology, Faridabad, Haryana, India

**Keywords:** TLRs, nucleoprotein VP2, innate immunity, ALSV, segmented flavivirus

## Abstract

The Alongshan virus (ALSV), a newly identified tick-borne segmented flavivirus, can infect humans and cause Alongshan fever, making it imperative to understand its pathogenic mechanisms for the development of effective intervention strategies. Our previous research has established that ALSV exhibits sensitivity to interferon-beta (IFN-β) while having evolved the ability to antagonize downstream antiviral responses induced by type I IFN (IFN-I); however, the specific effects and underlying mechanisms by which ALSV modulates IFN-I production remain poorly understood. In the present study, we demonstrated that ALSV infection significantly suppresses host IFN-I production triggered by poly(I:C), a synthetic analog of viral double-stranded RNA that activates innate immune pathways. To unravel the molecular basis of this suppression, we systematically evaluated the impact of individual ALSV viral proteins on Toll-like receptor-mediated IFN-I production, revealing a complex regulatory network wherein distinct viral proteins target specific signaling molecules: specifically, VP2 and VP3 were found to be responsible for inhibiting RIG-I-mediated IFN-I production, while NSP2 and VP1b were identified as key inhibitors of MDA5-mediated IFN-I production, highlighting the virus’s strategy of employing multiple proteins to disrupt innate immune signaling. Focusing further on the viral nucleoprotein VP2, we determined that it acts at the upstream signaling level of TANK-binding kinase 1, a critical kinase in the IFN-I signaling cascade. Mechanistically, VP2 directly interacts with RIG-I and mediates its degradation through an autophagy-dependent pathway, thereby impairing the host’s ability to detect viral RNA and initiate IFN-I production. These findings not only expand our understanding of the immune evasion mechanisms employed by novel segmented flaviviruses but also offer valuable insights that could facilitate the development of new preventive and therapeutic strategies for the ALSV infection.

**IMPORTANCE:**

Alongshan virus (ALSV) is an emerging segmented flavivirus that poses a growing threat to human and animal health across Eurasia. Despite its demonstrated capacity to infect humans and suppress interferon (IFN)-mediated antiviral responses, the precise mechanisms of ALSV immune evasion remain largely undefined. This study identifies the viral nucleoprotein VP2 as a key antagonist of host type I IFN (IFN-I) production. By directly interacting with and promoting the autophagy-mediated degradation of RIG-I, VP2 effectively disrupts innate immune recognition and signaling. This finding not only elucidates a previously unknown mechanism of immune suppression by ALSV but also highlights the virus’s sophisticated strategy of using multiple proteins to selectively target RIG-I and MDA5 pathways. These insights advance our understanding of segmented flavivirus-host interactions and suggest that restoring RIG-I function may be a promising therapeutic strategy against ALSV infection.

## INTRODUCTION

Emerging infectious diseases pose a serious threat to human health and economic development. With the development of high-throughput sequencing, a novel class of segmented flaviviruses has been discovered. The main members include Jingmen tick virus (JMTV), Mogiana tick virus, Alongshan virus (ALSV), and Guaico Culex virus (1, 2). Currently, the segmented flaviviruses are widely distributed across Asia, Africa, the Americas, and Europe. Their hosts include arthropods such as ticks and mosquitoes, as well as mammals such as monkeys, sheep, and cattle (3, 4). Notably, JMTV and ALSV can infect humans and cause diseases, thereby posing a significant public health threat (2, 5). ALSV, a newly discovered tick-borne segmented flavivirus, can infect humans and cause Alongshan fever, characterized primarily by fever, headache, and fatigue (2). Additionally, ALSV has been found to infect various wild animals, including reindeer, migratory birds, pandas, and brown rats, with viremia observed in reindeer (4, 6). So far, ALSV has been found in China, Finland, France, Russia, and other countries (7–9). As an emerging zoonotic virus, there are currently no drugs or vaccines available for ALSV, and it is urgent to conduct research on its pathogenesis.

Unlike traditional flaviviruses with a single linear genome, the genome of the segmented flaviviruses is divided into 4–5 segments. ALSV is a positive-sense single-stranded RNA virus with the genome divided into four segments (S1–S4) (2). S1 and S3, respectively, encode nonstructural proteins NSP1 and NSP2, which are homologous to the NS5 and NS2b3 of traditional flaviviruses, while S2 and S4 encode structural proteins: S2 encodes glycoproteins VP1a, VP1b, and VP4, and S4 encodes the capsid protein VP2 and the membrane protein VP3.

In all species, innate immune responses, especially the type I interferon (IFN-I) system, represent the first line of defense against viral infections. Upon viral entry, host pattern recognition receptors (PRRs) recognize pathogen-associated molecular patterns, initiating complex signal transduction cascades that ultimately induce the expression of antiviral effector genes (10). Viral-encoded immune antagonists specifically target input and output nodes of IFN signaling. These immune evasion capabilities are crucial not only for successful viral replication but also for determining host specificity and tissue tropism (11).

During co-evolution with hosts, viruses have developed multiple strategies to suppress IFN responses, including inhibition of IFN-I production and downstream signaling. Various flaviviral NSPs suppress IFN-I by targeting the RIG-I-like receptor (RLR) signaling pathway, for example, DENV NS2B3 blocks RIG-I mitochondrial translocation via 14-3-3ε binding (12); DENV NS2A/4B and ZIKV NS5 inhibit TANK-binding kinase 1 (TBK1) phosphorylation to suppress RIG-I/MDA5-induced IFN-I production (13, 14); and DENV NS4A and ZIKV NS3/4A competitively bind MAVS, blocking downstream signaling (14, 15). In previous studies, we found that ALSV is sensitive to IFN-β and evolves the ability to antagonize the IFN-I-induced downstream antiviral responses (16). Mechanistically, ALSV’s NSP1 interacts with and degrades human STAT2 through an autophagy pathway, directly inhibits the expression of IFN-stimulated genes (ISGs), or induces mitophagy to suppress the host’s innate immune (16). However, the effect and mechanism of ALSV on IFN-I production remain unclear.

In this study, we investigated the impact of ALSV-encoded proteins on IFN-I production and identified that the nucleoprotein VP2 significantly suppressed IFN-I production. Mechanistically, VP2 binds to and promotes the autophagic degradation of RIG-I, thus antagonizing the host innate immune response. These findings not only enrich the immune escape mechanism of the novel segmented flavivirus but also provide a new strategy for the prevention and control of ALSV infection.

## MATERIALS AND METHODS

### Cells and antibodies

Human embryonic kidney (HEK293T) cells (ATCC, cat# CRL-3216), A549 cells (Procell, cat# CL-0016), and T98G cells (Procell, cat# CL-0583) were grown in Dulbecco’s modified Eagle’s medium (DMEM, high glucose) (Sigma-Aldrich, cat# R8758-500ML), supplemented with 10% fetal bovine serum (FBS) (BBI, cat# E600001) and 1% antibiotics (penicillin and streptomycin, PS) (Sangon, cat# B540732). To induce an IFN-I response, HEK293T cells were transfected with poly(I:C) (Invivogen, Cat# tlrl-pic) for the specified durations.

The following primary antibodies were utilized: glyceraldehyde 3-phosphate dehydrogenase (GAPDH; ProteinTech, cat#10494-1-AP), HA (ProteinTech, cat#51064-2-AP), GST (ProteinTech, cat#10000-0-AP), Flag (ProteinTech, cat#20543-1-AP), Myc (ProteinTech, cat#60003-2-Ig), β-Actin (ProteinTech, cat#66009-1-Ig), IFIT3 (ProteinTech, cat#15201-1-AP), IFIT1 (Cell Signaling Technology, cat#14769), Phospho-IRF3 (Abways, cat#CY6575), Phospho-TBK1 (Cell Signaling Technology, cat#5483s), IRF3 (ProteinTech, cat#11312-1-AP), TBK1 (Abcam, cat#AB40676), RIG-I (ProteinTech, cat#20566-1-AP), MDA5 (ProteinTech, cat#21775-1-AP), MAVS (ProteinTech, cat#14341-1-AP), TRAF3 (ProteinTech, cat#18099-1-AP), LC3 (ProteinTech, cat#14600-1-AP), and ATG5 (HUABIO, cat#ET1611-38).

### Plasmids and transfection

The cDNAs corresponding to the viral protein sequences of ALSV were subcloned into the Xba I site of 3 × Flag-VR1012 expression vector as previously described (17). The promoter reporter plasmid pIFN-β-Fluc, which expresses firefly luciferase, along with the internal reference reporter plasmid pGL4.74, which expresses the Renilla Luciferase, GST-tagged RIG-IN (comprising the constitutively active N-terminal domains of RIG-I) (18, 19), HA-tagged RIG-I, TBK1, MDA5, and Myc-tagged IRF3 were obtained as previously described (20, 21). Additionally, Myc-MAVS was purchased from the Miaoling Plasmid Library (Wuhan, China).

All constructed plasmids were transformed into Stbl3 (Thermo Fisher Scientific, Cat# C737303) or Trans5α (TransGen, cat# CD201-01) chemically competent cells, followed by selection on antibiotic-containing LB agar plates. Transfection of the plasmids into the specified cells was performed using Lipofectamine 3000 (Invitrogen, San Diego, CA, USA) when the cells had grown to approximately 80%–90% confluence. For siRNA transfection, Lipofectamine RNAimax (Invitrogen, 13778100) was utilized following the manufacturer’s guidelines. The ATG5 siRNA and negative control siRNA were designed and synthesized in GENCEFE. The sequences were as follows: ATG5 siRNA1: sense: GGAAUAUCCUGCAGAAGAATT; anti-sense: UUCUUCUGCAGGAUAUUCCTT; ATG5 siRNA2: sense: GCAGAUGGACAGUUGCACATT; anti-sense: UGUGCAACUGUCCAUCUGCTT; ATG5 siRNA3: sense: AGAAUAUAUCAGACAACGATT; anti-sense: UCGUUGUCUGAUAUAUUCUTT.

### Virus and infection

ALSV was isolated from a patient who had been bitten by a tick in Northeast China (2). Working virus stocks of ALSV were prepared as previously described (22). All experiments involving infectious ALSV were conducted in strict compliance with Biosafety Level 2 conditions. Manipulations involving both inactivated and non-inactivated ALSV followed the guidelines and regulations set forth by the Chinese authorities regarding dual-use pathogens.

For viral infection, HEK293T cells were inoculated with ALSV virus stocks (MOI 2.5) and incubated at 37°C in serum-free DMEM (high glucose) for 2 h. The infected cells were subsequently washed twice with PBS and cultured in 300 μL of DMEM (high glucose) containing 2% FBS and 1% PS. For viral RNA quantification, the culture supernatant was harvested at the specific time points and subjected to Taqman-qPCR using ALSV segment 2 (S2)-specific primers: 5′-GCTTGTGGTCATCATTATG-3′ (forward), 5′-CTCTGCCACATACTGATG-3′ (reverse), and 5′-CTCTCGTCAGCCATACCACCA-3′ (probe primer) as previously described (22).

### Quantitative real-time PCR

Quantitative real-time PCR (qPCR) was performed using the primers listed in the Supplemental Table, following the established protocols (23). In brief, after the specified treatment as indicated in the Figure legends, cells were lysed using a lysis buffer. Total RNA was extracted using the EasyPure RNA Kit and subsequently reverse-transcribed into cDNA using the cDNA Synthesis SuperMix (TransGen, cat# AT341). For the qPCR step, Fast SYBR Green Master Mix (Roche, cat# 4913850001) was used, and the reactions were run on a Step-One Plus real-time PCR system. The GAPDH gene was employed as the reference gene for normalization.

### Dual luciferase reporter assay

The dual luciferase reporter assay was conducted as previously described (20). For the ALSV infection experiment measured via dual luciferase reporter assay, HEK293T cells were seeded in 24-well plates at a density of 2 × 10^5^ per well. They were co-transfected with 250 ng of the IFN-β-Luc and 50 ng of pGL4.74, which constitutively expresses the Renilla luciferase, along with 0.6 ng of poly(I:C). At 24 h post-transfection (hpt), the cells were infected with ALSV and incubated for either 24 or 48 h. Then the cells were harvested, and the luciferase activity was measured using the dual-luciferase reporter assay (Promega, cat# E1910). For experiments involving the transfection of viral proteins, HEK293T cells cultured in 24-well plates were co-transfected with 500 ng of the indicated plasmids expressing viral proteins, along with 250 ng of IFN-β-Luc and 50 ng of pGL4.74, along with the IFN-I activator expressing plasmid. At 48 hpt, cells were analyzed for reporter activity. Average firefly luciferase values were normalized to average Renilla luciferase values. Mock or empty vector-treated samples without IFN-I activation were set to 1, and each sample’s luciferase activity was standardized to this value.

### Immunoblotting

Immunoblotting was performed as previously described (22). HEK293T cells were transfected with the indicated viral plasmids. At 48 hpt, cells were lysed for 30 min using a lysis buffer supplemented with a protease inhibitor cocktail (Selleck, cat# B14002). To detect phosphorylated proteins, cells were lysed using the same lysis buffer, but with HaltTM protease and phosphatase inhibitor single-use cocktail (Thermo Fisher Scientific, cat# 78442). The cell lysates were then centrifuged at 12,000 × *g* for 10 min at 4°C, and the protein concentration was determined using Pierce BCA Protein Assay Kits (Thermo Fisher Scientific, cat# 23225). The lysates were subsequently reduced with 1 × Protein Loading buffer (TransGen, cat# DL101-02) for 5 min at 95°C. For electrophoresis, 20–30 μg of protein for each sample was separated by 8%–15% sodium dodecyl sulfate-polyacrylamide gel electrophoresis and transferred onto PVDF membranes (Millipore, cat# IPVH00010) using Trans-Blot Systems (Bio-Rad). Following transfer, the membranes were blocked with 2% BSA in PBST and then incubated with the appropriate primary antibody at 4°C overnight. After washing, the membranes were incubated with HRP-conjugated secondary antibodies, and antibody-antigen complexes were visualized using a chemiluminescence (ECL) substrate (Millipore, cat# WBKlS0100). The grayscale analysis of the bands was performed with ImageJ software (16).

For the RIG-I degradation inhibitor experiment, HEK293T cells transfected with Flag-tagged VP2 or vector plasmid were treated with the following compounds for 12 h: 10 μM of MG132 (Merck, Cat# 474787), 10 μM of chloroquine (CQ, MCE, Cat# HY-17589A), or 10 mM of 3-Methyladenine (3-MA, Selleck, Cat# S2767).

### Co-immunoprecipitation assays

The co-immunoprecipitation (co-IP) assays were performed as previously described (16). For the Co-IP assays, HEK293T cells were transfected with the specified plasmids. At 48 hpt, cell lysates were collected and centrifuged at 12,000 × *g* for 10 min at 4°C to obtain whole-cell extract. The remaining lysate was then incubated with either anti-Flag M2 Affinity Gel (Sigma-Aldrich, cat# A2220), anti-HA Affinity Gel (Millipore, cat# E6779), or anti-Myc Affinity Gel (Abmart, cat# M20030M) at 4°C overnight to facilitate the immunoprecipitation process. The binding beads were washed several times with lysis buffer and subsequently denatured in 1× protein loading buffer for 10 min. Finally, the proteins within immunocomplexes and whole-cell extracts were analyzed using immunoblotting with the appropriate antibodies.

### Assay of cytotoxicity

The HEK293T cells were placed in 96-well plates at a density of 3 × 10^3^ cells/well. Drugs were added to cells after cell growth at 24 h and incubated for 12 h. Then, the cells with CCK-8 reagent (Selleck, cat#B34304) were incubated for 1 h, and the absorbance at 450 nm was measured using a microplate reader. The 50% of the maximum growth inhibition (IC50) was calculated.

### Immunofluorescence

HEK293T cells cultured on 24-well plates were transfected with the indicated plasmids. After 48 h, cells were fixed with 4% paraformaldehyde and permeated with 0.5% Triton X-100. After cells were washed with PBST, they were blocked in 5% BSA and stained with primary antibodies, followed by staining with CoraLite 594 or 488-conjugated IgG secondary antibodies. Nuclei were stained with DAPI (Yesen, 40728ES03). Fluorescence images were obtained and analyzed using a confocal microscope (Nikon, AXR [Ti2-E]). Average fluorescence intensity and positive rate were analyzed using ImageJ.

### Quantification and statistical analysis

Data analyses were conducted using Prism 9.0.2 (GraphPad Software). The data are presented as the mean ± standard deviation (SD). Statistically significant differences from independent experiments (*n* ≥ 3) were determined by one- or two-way analysis of variance (ANOVA) with multiple comparisons correction. Significance is indicated by asterisks, as follows: *P < 0.05, **P < 0.01, ***P < 0.001, ****P < 0.0001.

## RESULTS

### ALSV-encoded proteins regulate the host’s type I IFN production

**Fig 1:**
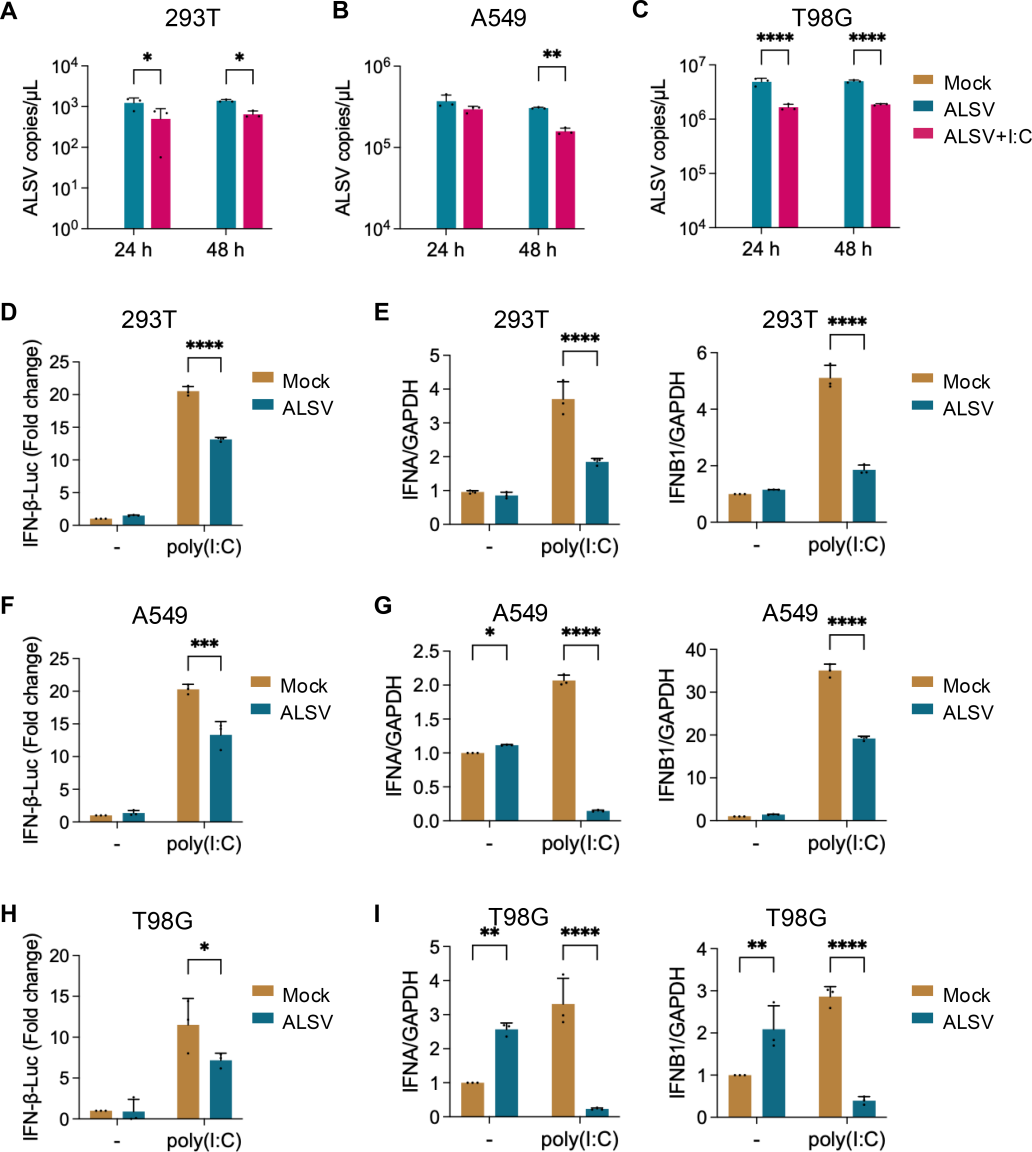
ALSV-encoded proteins regulate the host’s type I IFN production. (**A–C**) HEK293T, A549, and T98G cells were infected with ALSV. At 2 h post-infection (hpi), cells were transfected with or without poly(I:C) to activate IFN-I response. After 24 or 48 h, viral RNA copies in supernatants were quantified using TaqMan-qPCR. (**D**) HEK293T cells were co-transfected with an IFN-β-luc plasmid and a control plasmid pGL4.74, along with poly(I:C). At 24 hpt, cells were infected with ALSV. At 24 hpi, cells were harvested, and luciferase activity was measured. (**E**) HEK293T cells transfected with poly(I:C) were mock-infected or infected with ALSV. At 48 hpi, the mRNA levels of host *IFNA* and *IFNB1* were examined using qPCR. (**F**) A549 cells were treated as indicated in (**D**), and luciferase activity was measured. (**G**) A549 cells were treated as indicated in (**E**). At 48 hpi, the mRNA levels of *IFNA* and *IFNB1* were examined using qPCR. (**H**) T98G cells were treated as indicated in (**D**), and luciferase activity was measured. (**I**) T98G cells were treated as indicated in (**E**). At 48 hpi, the mRNA levels of *IFNA* and *IFNB1* were examined using qPCR. Data from independent experiments (*n* ≥ 3) were statistically analyzed using one- or two-way ANOVA with multiple comparison correction (*P < 0.05, **P < 0.01, ***P < 0.001, and ****P < 0.0001).

In previous studies, we found that ALSV is sensitive to IFN-I antiviral response and evolves the ability to antagonize the IFN-I-induced downstream antiviral responses (16). Poly(I:C) is a synthetic double-stranded RNA (dsRNA) molecule that mimics viral genetic material. It is recognized by Toll-like receptor 3 (TLR3 in endosomes) and RLRs (including RIG-I and MDA5 in the cytoplasm) pathways, leading to the production of type I IFNs (IFN-α/β) and pro-inflammatory cytokines (24). Here, we also found that poly(I:C)-induced IFN-I response restrained the ALSV replication in multiple cell lines, including HEK293T (human embryonic kidney cells), A549 cells (human lung epithelial cells), and T98G cells (human glial cells) (Fig. 1A through C), and ALSV infection inhibited the *IFIT2*, *IFIT3*, and *IRAV* of ISGs expression induced by poly(I:C) in these cells (Fig. S1). The immunofluorescence analysis confirmed that ALSV infection attenuated poly(I:C)-induced upregulation of IFIT1/IFIT3 proteins (Fig. S2) and suppressed poly(I:C)-triggered phosphorylation of IRF3 (Fig. S3A). However, the effect of ALSV on IFN-I production remains unclear. To determine whether ALSV inhibits the host’s IFN-I production, we co-transfected HEK293T, A549, and T98G cells with an IFN-β luciferase reporter plasmid and the control plasmid pGL4.74, along with poly(I:C) to induce the IFN-I production, followed by infection with ALSV (MOI = 2.5). The results showed that ALSV infection significantly suppressed poly(I:C)-induced IFN-β promoter activity (Fig. 1D, F, and H). Additionally, ALSV infection markedly reduced the mRNA expression of *IFNA* and *IFNB1* (Fig. 1E, G, and I), suggesting that ALSV inhibits IFN-I production and thereby suppresses the host antiviral innate immune response.

To investigate the potential mechanism by which ALSV suppresses IFN-I production, we initially examined the regulatory impact of viral proteins on poly(I:C)-induced IFN-I production. Specifically, HEK293T cells were co-transfected with viral proteins and poly(I:C). Immunoblotting verified the successful expression of all ALSV proteins, and the luciferase assay demonstrated that NSP2, VP1a, VP2, and VP4 significantly repressed poly(I:C)-induced IFN-β promoter activation (Fig. S3B). Furthermore, qPCR results corroborated that NSP2, VP2, and VP4 also inhibited the transcription of *IFNA*, *IFNB1*, as well as *OAS1* of ISGs (Fig. S3C). Overall, these data suggest that ALSV infection can impede IFN-I production, potentially mediated by the viral proteins NSP2, VP2, and VP4.

### ALSV proteins VP2 and VP3 inhibit the RIG-I-induced IFN-I production

**Fig 2:**
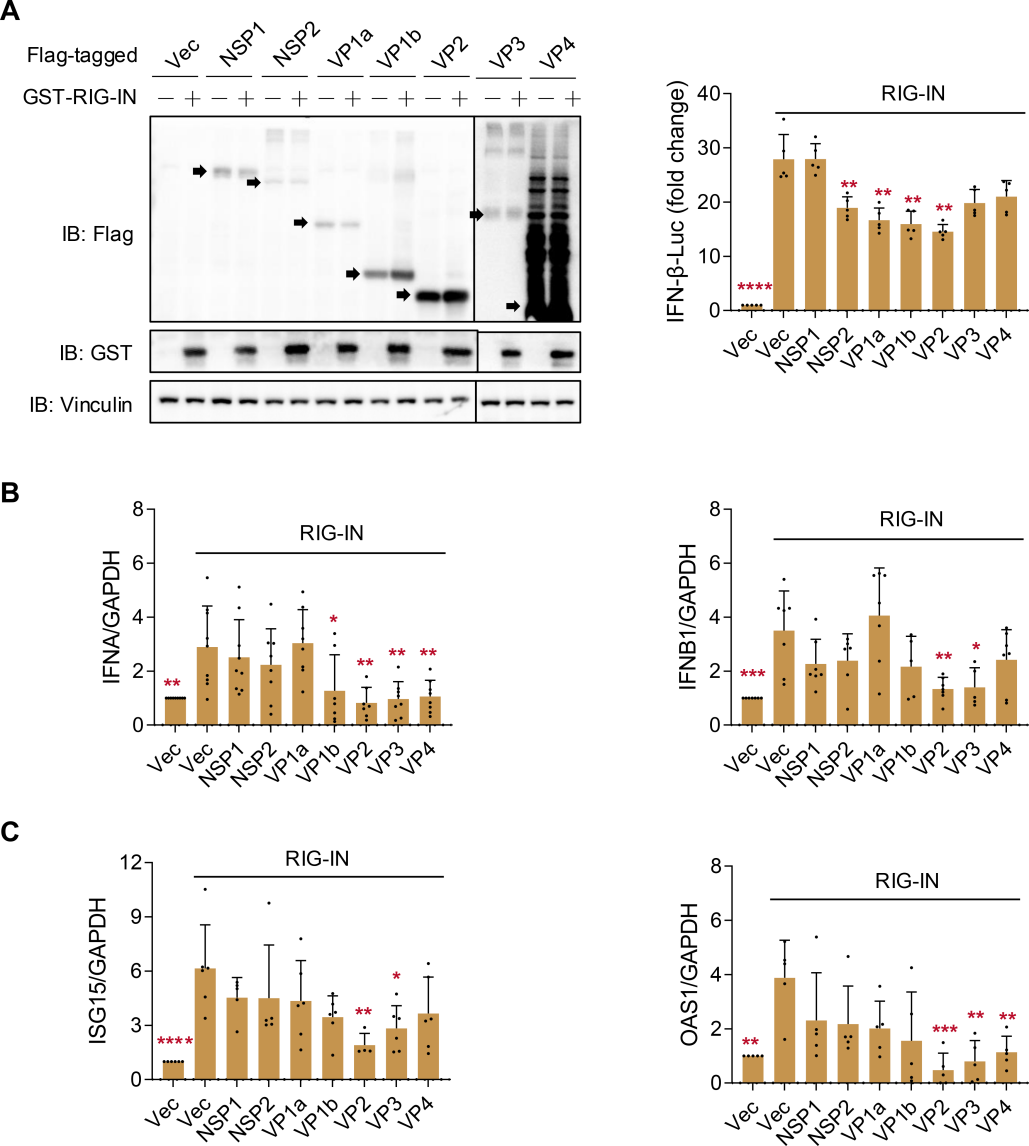
ALSV proteins regulate RIG-I-induced IFN-I production. (**A**) HEK293T cells were transfected with an IFN-β-luc reporter plasmid, a control plasmid, and plasmids expressing ALSV proteins, along with GST-RIG-IN to induce IFN-I production. At 24 hpt, cells were subjected to immunoblotting and luciferase activity assays. (**B and C**) HEK293T cells were transfected with GST-RIG-IN and plasmids expressing ALSV proteins. At 24 hpt, the mRNA levels of host *IFNA*, *IFNB1* (**B**), and *ISG15*, *OAS1* (**C**) were examined using qPCR, with *GAPDH* serving as the internal reference control. Statistical analysis was performed on data from independent experiments (*n* ≥ 3), with comparisons to the RIG-IN-activated Vector group using one-way ANOVA followed by multiple comparison correction (*P < 0.05, **P < 0.01, ***P < 0.001, and ****P < 0.0001).

Subsequently, we investigated the effects of ALSV viral proteins on the IFN-I response induced by the RLRs. First, we evaluated the classical RIG-I-like receptor (RIG-I)-induced signaling. HEK293T cells were co-transfected with plasmids expressing ALSV proteins and RIG-IN, which consists of the constitutively active N-terminal domains of RIG-I. Immunoblotting confirmed the successful expression of all ALSV proteins and the RIG-IN protein. The luciferase assay indicated that NSP2, VP1a, VP1b, and VP2 significantly inhibited RIG-I-induced IFN-β promoter activation (Fig. 2A). Additionally, qPCR showed that VP2 and VP3 downregulated the expression of *IFNA* and *IFNB1*, as well as the ISGs genes *ISG15* and *OAS1* (Fig. 2B and C). In summary, these results suggest that the ALSV proteins VP2 and VP3 may be responsible for the inhibition of RIG-I-induced IFN-I production.

### ALSV proteins NSP2 and VP1b inhibit MDA5-induced IFN-I production

**Fig 3:**
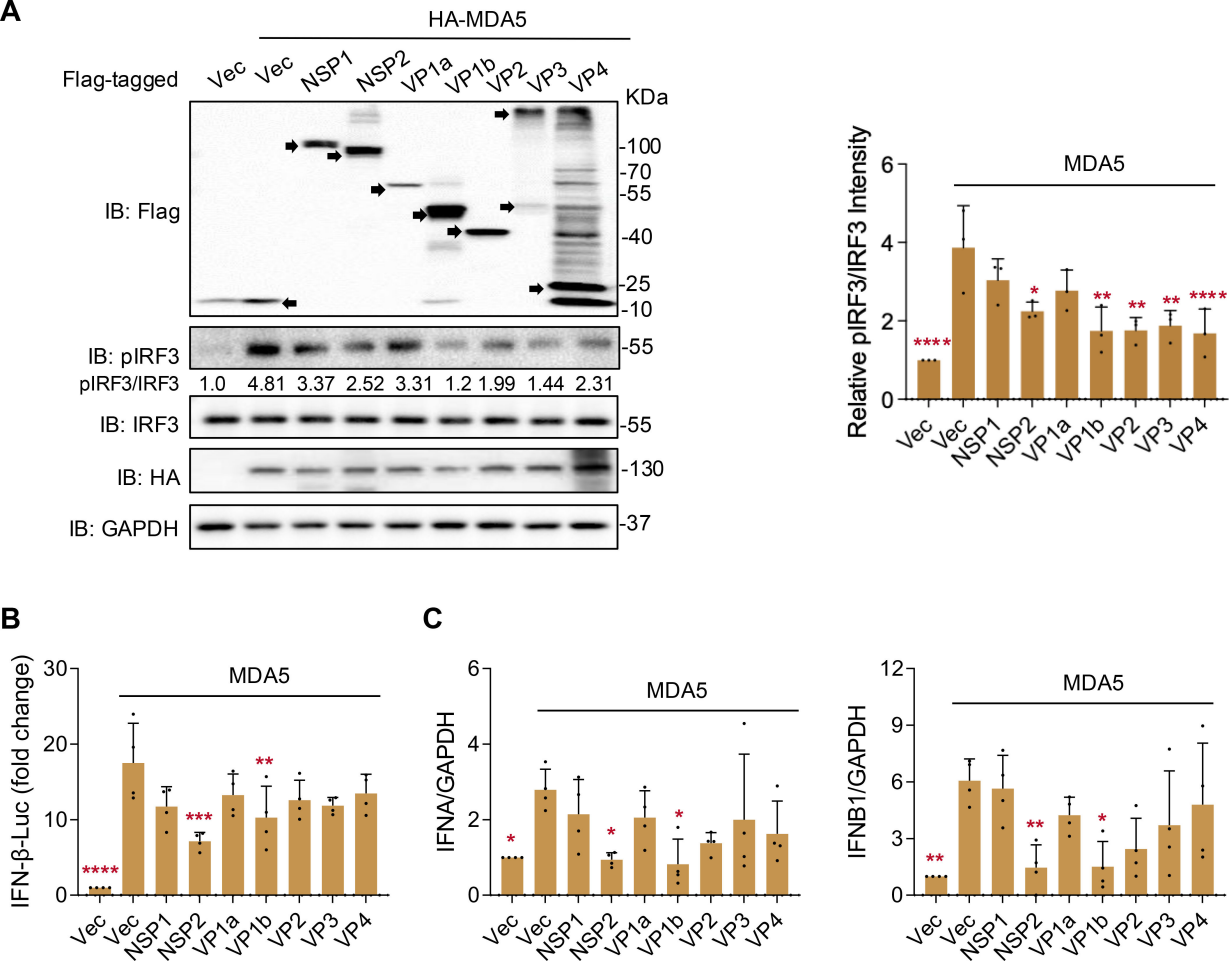
ALSV proteins regulate MDA5-induced IFN-I production. (**A**) HEK293T cells were transfected with an IFN-β-luc reporter plasmid, a control plasmid, and plasmids expressing ALSV proteins, along with HA-MDA5 to induce IFN-I production. At 24 hpt, cells were subjected to immunoblotting, and the relative levels of phosphorylated IRF3 normalized to IRF3 are shown in the right. (**B**) HEK293T cells were treated as indicated in (**A**), and cells were subjected to luciferase activity assays. (**C**) HEK293T cells were transfected with HA-MDA5 and plasmids expressing ALSV proteins. At 24 hpt, the mRNA levels of host *IFNA* and *IFNB1* were examined using qPCR, with *GAPDH* serving as the internal reference control. Statistical analysis was performed on data from independent experiments (*n* ≥ 3), with comparisons to the MDA5-activated Vector group using one-way ANOVA followed by multiple comparison correction (*P < 0.05, **P < 0.01, ***P < 0.001, and ****P < 0.0001).

Furthermore, we assessed whether MDA5-induced signaling, another member of the RIG-I receptors, is disrupted by ALSV proteins. The immunoblotting showed that various viral proteins inhibited the phosphorylation of IRF3, a downstream signaling molecule of MDA5, including NSP2, VP1b, VP2, VP3, and VP4 (Fig. 3A). The luciferase assay indicated that NSP2 and VP1b significantly repressed MDA5-induced IFN-β promoter activation (Fig. 3B), and qPCR confirmed the suppression of *IFNA* and *IFNB1* transcription by NSP2 and VP1b (Fig. 3C). However, although the VP2 protein reduced MDA5-induced IFN-β promoter activity and *IFNA* and *IFNB1* transcription, this difference was not statistically significant. This suggests that VP2 inhibits IFN-I production mainly by acting on RIG-I molecules rather than the downstream signaling molecules of RLRs. In summary, these results imply that the ALSV proteins NSP2 and VP1b may be responsible for the inhibition of MDA5-induced IFN-I production.

### ALSV VP2 suppresses IFN-I production by targeting the upstream of TBK1

**Fig 4:**
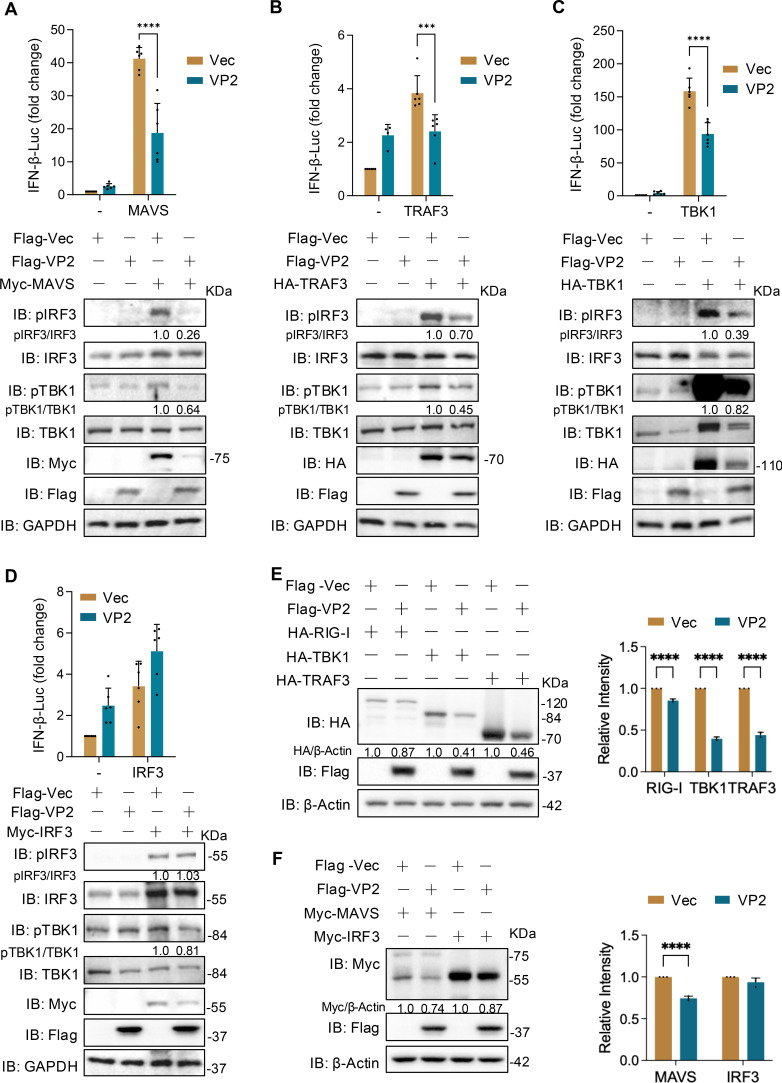
ALSV VP2 suppresses IFN-I production by targeting the upstream of TBK1. (**A–D**) HEK293T cells were transfected with an IFN-β-luc reporter plasmid, a control plasmid, and plasmids expressing ALSV VP2 protein, along with MAVS (**A**), TRAF3 (**B**), TBK1 (**C**), and IRF3 (**D**) expressing plasmids to induce IFN-I production. At 24 hpt, cells were subjected to the luciferase activity assay and immunoblotting analysis using the phosphorylation and total antibodies of IRF3 and TBK1, along with the tag antibodies. Gray-scale statistical analysis of phosphorylation relative to total protein is conducted. (**E and F**) HEK293T cells were transfected with plasmids expressing ALSV VP2 protein, along with the indicated signaling molecules. At 48 hpt, cells were subjected to immunoblotting analysis. The relative levels of the indicated proteins normalized to β-Actin are shown. Data from independent experiments (*n* ≥ 3) were statistically analyzed using two-way ANOVA with multiple comparison correction (***P < 0.001 and ****P < 0.0001).

Given the crucial role of VP2 in counteracting the host’s antiviral response mediated by IFN-I, we further concentrated on analyzing the specific molecular mechanism behind its inhibition of IFN-I production. HEK293T cells were co-transfected with plasmids expressing VP2 and downstream signaling molecules of RLRs (MAVS, TRAF3, TBK1, IRF3). At 24 hpt, the cells were subjected to luciferase activity and immunoblotting analysis. The results indicated that VP2 significantly suppressed MAVS-, TRAF3-, and TBK1-induced IFN-β promoter activity as well as TBK1/IRF3 phosphorylation but did not influence IRF3-induced activation (Fig. 4A through D). Furthermore, we explored VP2’s effect on protein levels and found that VP2 reduced the protein levels of RIG-I, TBK1, TRAF3, and MAVS, but not IRF3 (Fig. 4E and F), implying that VP2 acts on the upstream signal molecule of TBK1.

### ALSV VP2 interacts with RIG-I and TBK1

**Fig 5:**
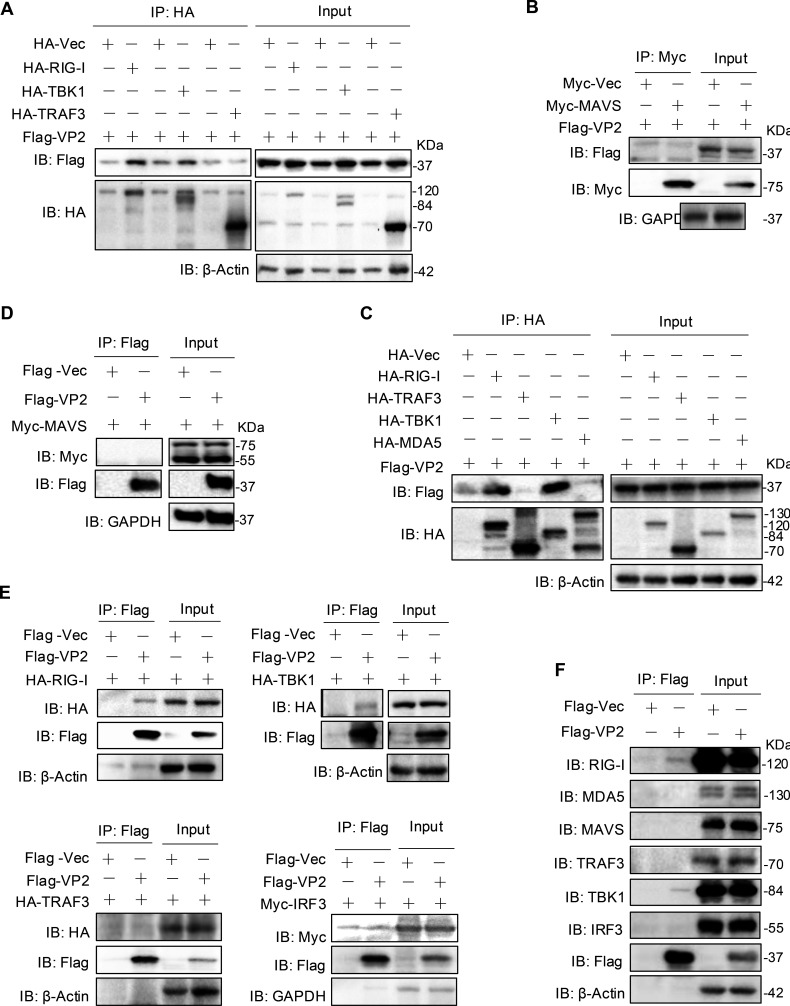
ALSV VP2 interacts with RIG-I to suppress its activity. (**A**) HEK293T cells were transfected with Flag-VP2 and HA-tagged RIG-I, TBK1, TRAF3, or an empty vector. At 48 hpt, cells were subjected to anti-HA immunoprecipitates and analyzed by immunoblotting. (**B**) HEK293T cells were transfected with Flag-VP2 and Myc-tagged MAVS or an empty vector. At 48 hpt, cells were subjected to anti-Myc immunoprecipitates and analyzed by immunoblotting. (**C**) HEK293T cells were transfected with Flag-VP2 and HA-tagged RIG-I, TBK1, TRAF3, MDA5, or an empty vector. At 48 hpt, anti-HA immunoprecipitates were analyzed by immunoblotting. (**D**) HEK293T cells were transfected with Flag-VP2 and Myc-tagged MAVS or an empty vector. At 48 hpt, cells were subjected to anti-Flag immunoprecipitates and analyzed by immunoblotting. (**E**) HEK293T cells were transfected with Flag-VP2, along with HA-RIG-I, HA-TBK1, HA-TRAF3, or Myc-IRF3. At 48 hpt, cells were subjected to anti-Flag immunoprecipitates and analyzed by immunoblotting. (**F**) HEK293T cells were transfected with Flag-VP2 or an empty vector. At 48 hpt, anti-Flag immunoprecipitates were analyzed by immunoblotting with the indicated endogenous antibodies.

To further identify the VP2-targeted IFN-I signaling molecules, we identified the signaling molecules interacting with VP2 by co-IP. HEK293T cells were transfected with RIG-I, MDA5, MAVS, TRAF3, TBK1, or IRF3, along with VP2 expressing plasmids. The co-IP results showed that only the RIG-I and TBK1 interacted with VP2, but not the MAVS, TRAF3, and IRF3 (Fig. 5A through E), indicating VP2 may directly target RIG-I and TBK1 to inhibit IFN-I production. Moreover, we further found that VP2 interacted with the endogenous RIG-I and TBK1, but not endogenous MDA5, MAVS, TRAF3, and IRF3 (Fig. 5F). Taken together, these results indicated that VP2 likely inhibits IFN-I production through interacting with RIG-I and TBK1.

### ALSV VP2 promotes RIG-I degradation via autophagy

**Fig 6:**
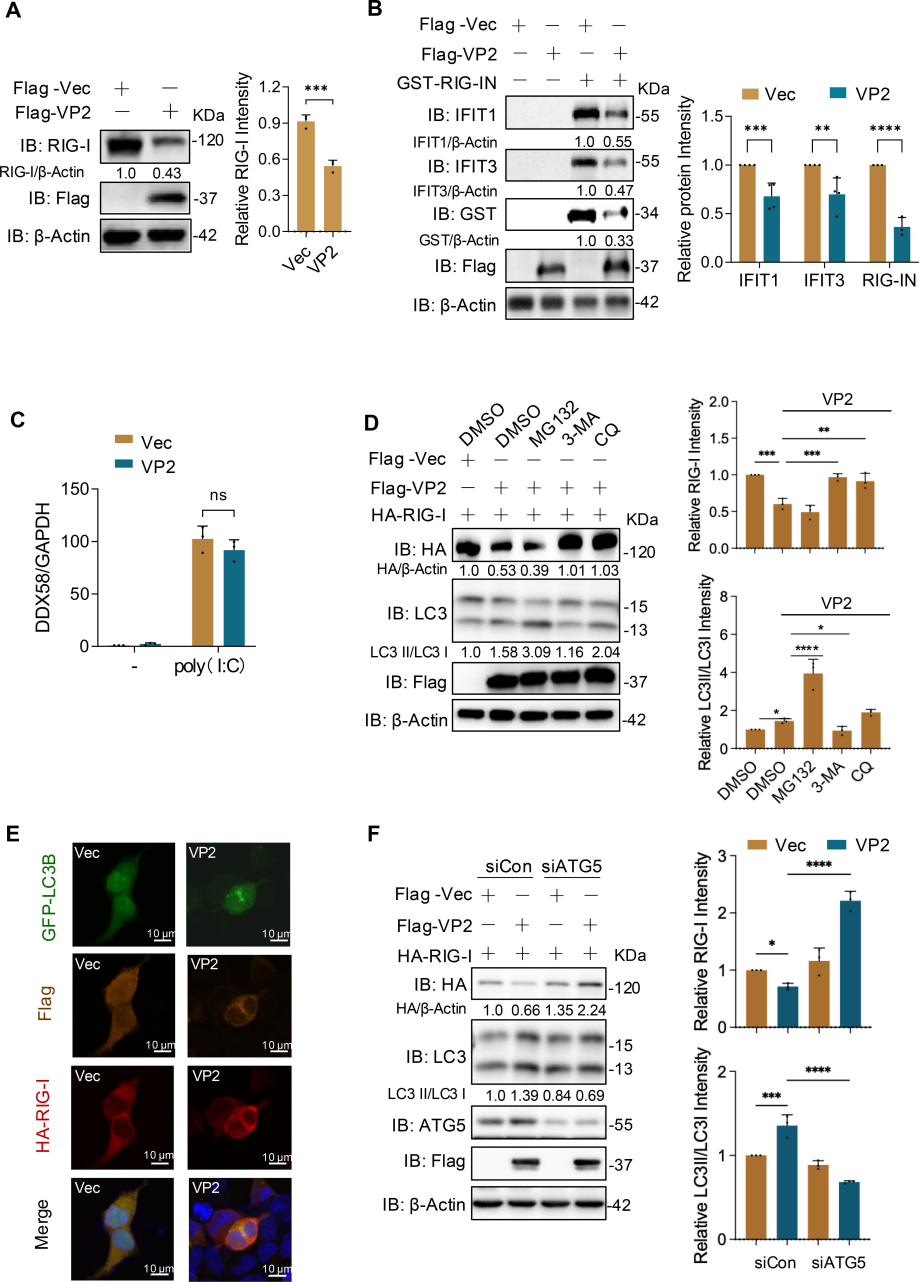
ALSV VP2 promotes RIG-I degradation via autophagy. (**A**) HEK293T cells were transfected with Flag-VP2 or an empty vector. At 48 hpt, cells were analyzed by immunoblotting with RIG-I antibody. Gray-scale statistical analysis of RIG-I relative to β-Actin is displayed on the right. (**B**) HEK293T cells were transfected with Flag-VP2, along with or without GST-RIG-IN. At 48 hpt, cells were analyzed by immunoblotting with the IFIT1 and IFIT3 antibodies. Gray-scale statistical analysis of IFIT1, IFIT3, and RIG-I-N relative to β-actin is displayed on the right. (**C**) HEK293T cells were transfected with Flag-VP2 or vector, along with poly(I:C). At 24 hpt, the mRNA levels of host DDX58 were examined using qPCR, with GAPDH serving as the internal reference control. (**D**) HEK293T cells were co-transfected with VP2 and RIG-I plasmids. At 24 hpt, cells were treated with the inhibitors MG132 (10 μM), CQ (10 μM), and 3-MA (10 mM) for 12 h. The cell lysates were analyzed by immunoblotting. (**E**) HEK293T cells were co-transfected with GFP-LC3B (Green), Flag-tag (Orange), and HA-RIG-I (Red) plasmids for 48 h and subjected to immunofluorescence staining. Nuclei were stained with DAPI. Scale bars, 10 µm. (**F**) HEK293T cells were transfected with siCon and siATG5 and cultured for 24 h, then co-transfected with VP2 and RIG-I plasmids for 24 h. The cells were analyzed by immunoblotting. Gray-scale statistical analysis of RIG-I relative to β-Actin is displayed on the right. Data from independent experiments (*n* ≥ 3) were statistically analyzed using one- or two-way ANOVA with multiple comparison correction (*P < 0.05; **P < 0.01; ***P < 0.001; ****P < 0.0001 and ns, not significant).

Furthermore, we found that VP2 significantly inhibited the expression of endogenous RIG-I and markedly decreased the RIG-I-N-induced expression of IFIT1 and IFIT3 (Fig. 6A and B). However, the qPCR results revealed that RIG-I (gene name *DDX58*) mRNA level was unaffected by VP2 (Fig. 6C), suggesting that VP2 did not affect the transcription of RIG-I. Given the interaction between VP2 and RIG-I (Fig. 5), we speculate that VP2 may reduce RIG-I protein by protein degradation. To validate using proteasome inhibitors, we first calculated the IC₅₀ of proteasome inhibitor MG132 (40.25 μM), chloroquine (CQ, 70.16 μM), and 3-MA (26.03 mM) using CCK-8 assays (Fig. S4A). HEK293T cells co-transfected with VP2 and RIG-I were treated with DMSO, MG132, or autophagy inhibitors CQ and 3-MA for 12 h. The results revealed that the autophagy inhibitors CQ and 3-MA, but not the proteasome inhibitor MG132, reversed the degradation of RIG-I caused by VP2 (Fig. 6D). Additionally, 3-MA reversed VP2-induced RIG-I degradation in a dose-dependent manner and reduced the LC3-II/LC3-I ratio (a marker of autophagosome initiation, which 3-MA inhibits) (Fig. S4B). Conversely, CQ increased LC3-II/LC3-I (by blocking autophagosome-lysosome fusion) but exerted a non-dose-dependent reversal of VP2-induced RIG-I reduction (Fig. S4C), potentially due to off-target signaling interference at high doses.

Besides, VP2 led to a marked increase in LC3B puncta, indicating activation/accumulation of autophagosomes (Fig. 6E). Additionally, inhibition of the autophagy pathway by downregulating ATG5 also reversed VP2-mediated RIG-I degradation (Fig. 6F; Fig. S4D). These results indicated that VP2 binds to RIG-I and mediates its degradation in an autophagy-dependent manner, thereby suppressing host antiviral defenses.

## DISCUSSION

Due to climate change, globalization, and urbanization, emerging viruses pose a continuous threat to global health. As contact between wildlife and humans increases, spillover events involving arthropod-borne viruses are becoming increasingly frequent (25). ALSV, a tick-borne zoonotic pathogen, belongs to the *Flaviviridae* family, *Jingmenvirus* group. Hard ticks serve as their vectors, while livestock such as cattle and sheep are their primary hosts (2, 9). However, the relevance of these factors to ALSV pathogenesis remains unclear and warrants further investigation. Our study shows that ALSV infection suppresses host IFN-I production, with multiple viral proteins having effects by targeting different signaling molecules. Subsequently, we focused on the viral nucleoprotein VP2, found that VP2 interacted with RIG-I and mediated its degradation in an autophagy-dependent manner, thus inhibiting IFN-I production and ISGs expression.

Tick-borne flaviviruses usually exhibit one of two clinical phenotypes: hemorrhagic fever or encephalitic disease. The pathogenicity of these viruses in humans, such as neuroinvasiveness and neurotoxicity, is mediated by virulence factors (26, 27). These virulence factors include structural proteins, NSPs, and the untranslated regions of the viral mRNA. These components play crucial roles in viral entry, replication, antigenicity, and immune evasion. DENV NSP NS2B3 prevents RIG-I translocation to mitochondria by mediating NS3’s binding with 14-3-3ε (12). DENV NS2A/4B and Zika virus NS5 inhibit RIG-I/MDA5-dependent IFN-I production by blocking TBK1 phosphorylation (13, 14). The DENV NS4A and Zika virus NS3/4A competitively bind MAVS with RIG-I or MDA5 to suppress downstream signaling pathways (14, 15). The Japanese encephalitis virus (JEV) NSP, NS1, suppresses MAVS expression through microRNA-22 (28). The Zika virus NS5 either inhibits RIG-I K63 chain ubiquitination by its methyltransferase (MTase) activity (29) or blocks downstream IRF3 phosphorylation via interaction with protein kinase IKKε (30). Here, we examined the regulatory impact of ALSV viral proteins on TLRs-induced IFN-I production, potentially mediated by the viral proteins NSP2, VP2, and VP4. The specific manifestations are as follows: VP2 and VP3 are responsible for the inhibition of RIG-I-induced IFN-I production, and NSP2 and VP1b are responsible for the inhibition of MDA5-induced IFN-I production.

As a classic TLR family PRR, RIG-I plays a key role in recognizing RNA virus infection and activating downstream innate immune antiviral response (31). However, during host-virus co-evolution, viruses have developed multiple strategies to modulate RIG-I-mediated IFN-I signaling. For instance, in positive-sense single-stranded RNA viruses, HCV encodes the NS3/4A protease, which disrupts RIG-I signaling by targeting Riplet (32); the ORF9-encoded N protein of SARS-CoV interacts with TRIM25 and inhibits RIG-I activation through impairing its ubiquitination (33); DENV prevents TRIM25-induced RIG-I signaling by deubiquitinating K48-linked chains on TRIM25 (34); and SFTSV NS protein associates with RIG-I and TRIM25 to inhibit the K63-linked ubiquitination of RIG-I (35, 36). In this study, we found that the ALSV VP2 protein directly interacts with RIG-I and promotes its degradation via the autophagy pathway, thereby suppressing IFN-I production. Autophagy is a conserved catabolic process that plays antiviral roles during viral infection. However, the co-evolution and adaptation between viruses and host autophagy mechanisms have enabled many viruses to hijack and manipulate autophagy to counteract host antiviral responses (37–39). However, the receptors and E3 ubiquitin ligases involved in RIG-I’s autophagic degradation mediated by ALSV VP2 protein remain unclear and will be further investigated in our future studies.

Our study also revealed that TBK1 interacts with ALSV VP2, suggesting that TBK1 may represent another target through which ALSV suppresses IFN-I production. Viruses have evolved diverse strategies to inhibit IFN-I responses by targeting TBK1. Some viral proteins suppress TBK1 activity or interfere with innate immune signaling complex formation. For example, Pseudorabies virus (PRV)-encoded UL13 protein degrades PRDX1 to block TBK1-IKKε interactions (40); African swine fever virus (ASFV)-encoded pS273R disrupts TBK1-IRF3 interaction, preventing IRF3 phosphorylation; and vimentin disrupts the TBK1-IKKε-IRF3 axis to inhibit IFN induction (41). On the other hand, viruses may also manipulate TBK1 post-translational modifications. For instance, ASFV A137R, MGF110-9L, and MGF505-7R proteins mediate TBK1 degradation via the autophagy-lysosome pathway (42); EBV deubiquitinase BPLF1 inhibits IFN-I production by regulating K63 and K48 ubiquitination of TBK1 (43); and Enterovirus 71 (EV71) promotes USP24 recruitment to TBK1 to remove K63-linked ubiquitin chains, suppressing IFN-I signaling (44). However, the mechanism by which ALSV nucleoprotein VP2 hijacks TBK1 to antagonize IFN-I response will be further elucidated in our future study.

In conclusion, we revealed that ALSV infection suppresses host IFN-I production, with multiple viral proteins having effects by targeting different signaling molecules. Mechanistically, ALSV nucleoprotein VP2 interacted with RIG-I and mediated its degradation in an autophagy-dependent manner, thus inhibiting IFN-I production and ISGs expression. This suggests that targeting RIG-I signaling may serve as a promising antiviral strategy against ALSV infection. Our findings elucidate the complex interplay between ALSV and host innate immunity and lay a foundation for future research and therapeutic development targeting emerging segmented flaviviruses.

## SUPPLEMENTAL MATERIAL

Figures S1 to S4 spectrum.02484-25-s0001.docx Fig. S1: ALSV suppresses poly(I:C)-induced ISGs expression. Fig. S2: ALSV attenuates poly(I:C)-induced IFIT1 and IFIT3 expression. Fig. S3: ALSV and its viral proteins suppress type I IFN response. Fig. S4: Blocking autophagy could attenuate the reduction of RIG-I induced by VP2.

Table S1 spectrum.02484-25-s0002.xlsx Primer pairs used for quantitative real-time PCR (qPCR).

ASM does not own the copyrights to Supplemental Material that may be linked to, or accessed through, an article. The authors have granted ASM a non-exclusive, world-wide license to publish the Supplemental Material files. Please contact the corresponding author directly for reuse.
