# Supplementary material for: The segmented flavivirus ALSV-encoded nucleoprotein VP2 inhibits type I interferon production by targeting RIG-I
Source: Microbiol Spectr. 2026 Jan 28;14(3):e02484-25. doi: 10.1128/spectrum.02484-25 (PMC12955381; doi:10.1128/spectrum.02484-25)
Supplement: Figures S1 to S4 — Fig. S1: ALSV suppresses poly(I:C)-induced ISGs expression. Fig. S2: ALSV attenuates poly(I:C)-induced IFIT1 and IFIT3 expression. Fig. S3: ALSV and its viral proteins suppress type I IFN response. Fig. S4: Blocking autophagy could attenuate the reduction of RIG-I induced by VP2. [file spectrum.02484-25-s0001.docx]

Supplementary Materials for

**The segmented flavivirus ALSV encoded nucleoprotein VP2 inhibits type I interferon production by targeting RIG-I**


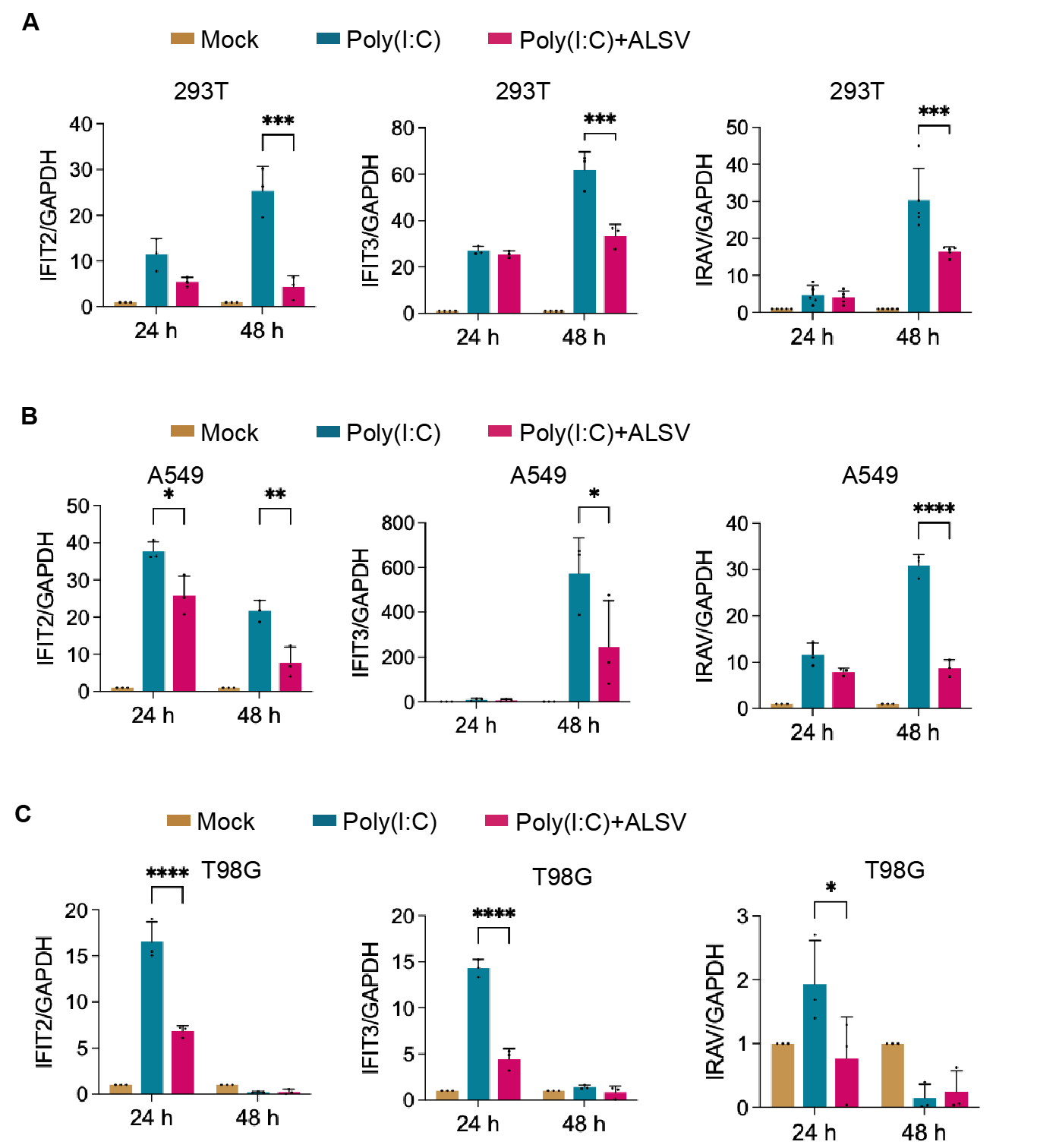


**Fig. S1 ALSV suppresses poly(I:C)-induced ISGs expression.** (A) HEK293T cells transfected with poly(I:C) were mock-infected or infected with ALSV. At 24 or 48 hpi, the mRNA levels of host *IFIT2*, *IFIT3*, and *IRAV* were examined using qPCR, with GAPDH serving as the internal reference control. (B-C) A549 and T98G cells were treated the same with HEK293T cells. The mRNA levels of host *IFIT2*, *IFIT3*, and *IRAV* were examined using qPCR. Data from independent experiments (n ≥ 3) were statistically analyzed using one- or two-way ANOVA with multiple comparison correction (**P* < 0.05, ***P* < 0.01, ****P* < 0.001, and *****P* < 0.0001).


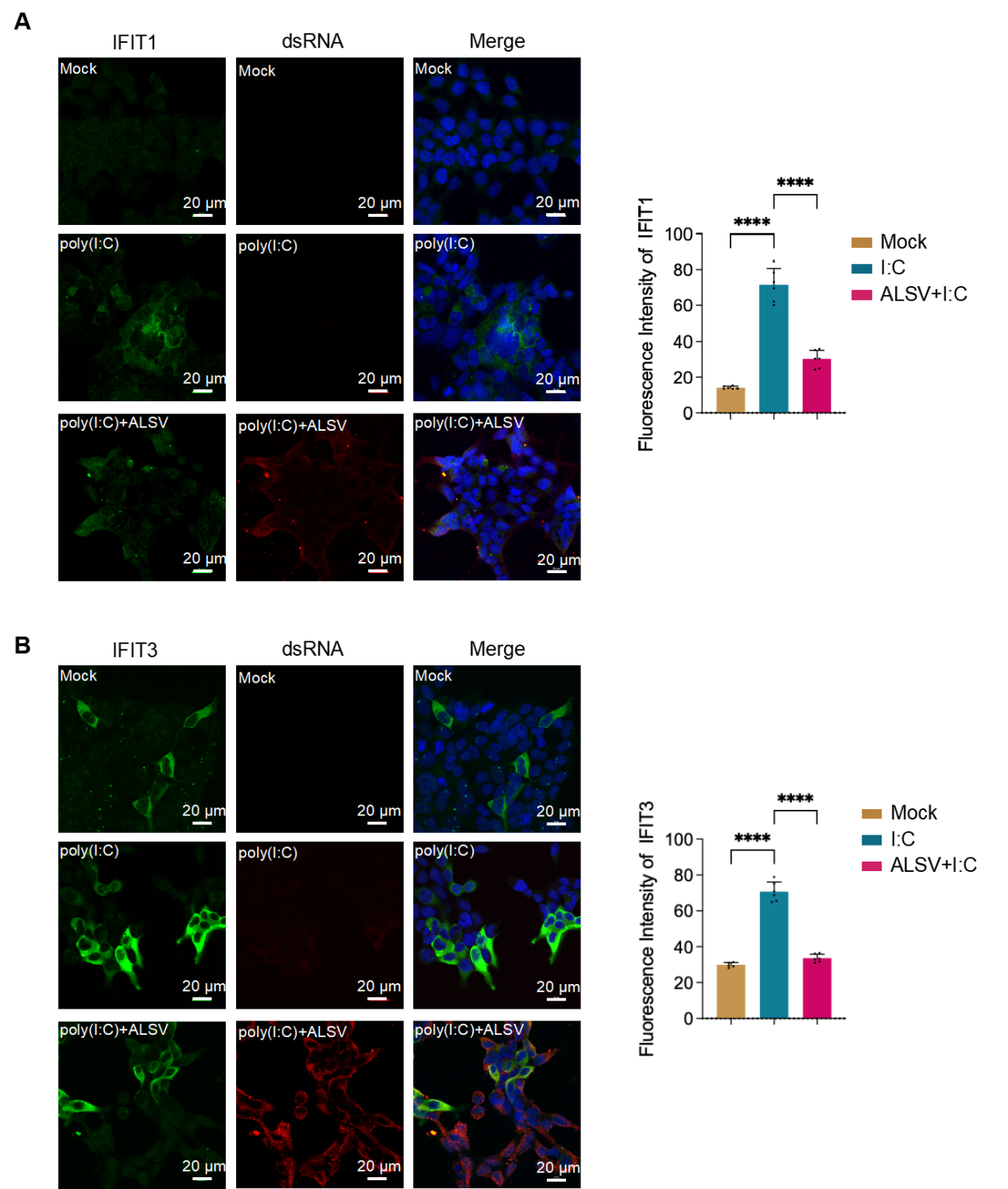


**Fig. S2 ALSV attenuates poly(I:C)-induced IFIT1 and IFIT3 expression.** (A, B) HEK293T cells transfected with poly(I:C) were mock-infected or infected with ALSV. At 48 hpi, the cells were subjected to immunofluorescence staining. IFIT1 (A) or IFIT3 (B) is shown in green, viral dsRNA is shown in red, and nuclei are stained with DAPI (blue). Scale bars, 20 μm. Quantification of mean cellular IFIT1 fluorescence intensity (mean ± SD, n = 3 independent experiments) is shown on the right. Statistical significance was assessed by one-way ANOVA with post-hoc multiple comparisons; *****P* < 0.0001 as indicated.


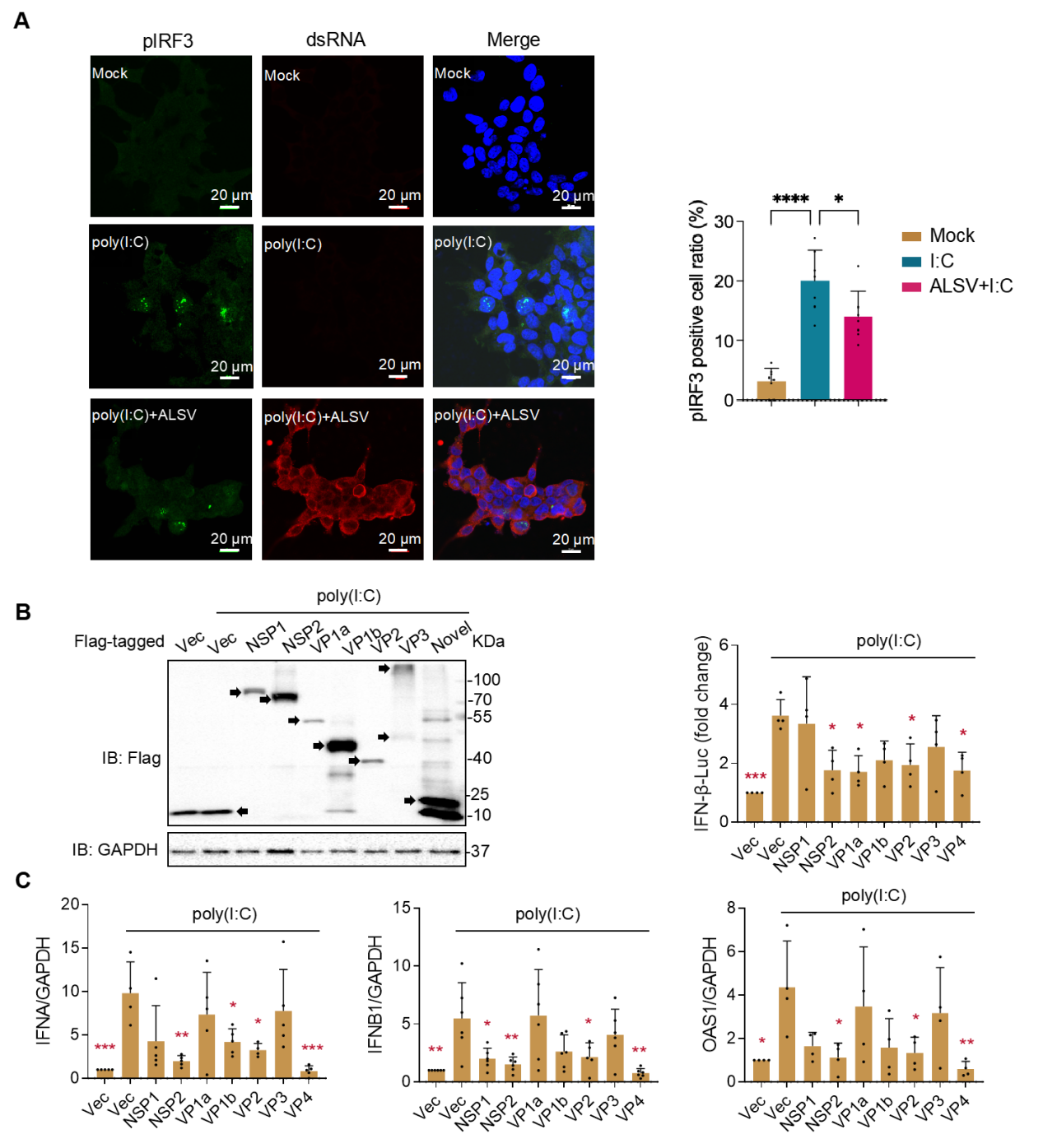


**Fig. S3 ALSV and its viral proteins suppress type I IFN response.** (A) HEK293T cells transfected with poly(I:C) were mock-infected or infected with ALSV. At 48 hpi, the cells were subjected to immunofluorescence staining. Phosphorylated IRF3 (pIRF3) is shown in green, viral dsRNA is shown in red, and nuclei are stained with DAPI (blue). Scale bars, 20 μm. The positive cells proportion for phosphorylated IRF3 is shown on the right. (B) HEK293T cells were transfected with an IFN-β-luc reporter plasmid, a control plasmid, and plasmids expressing ALSV proteins, along with poly(I:C). At 24 hpt, cells were subjected to immunoblotting and luciferase activity assays. (C) HEK293T cells were transfected with poly(I:C) and plasmids expressing ALSV proteins. At 24 hpt, the mRNA levels of host *IFNA*, *IFNB1*, and *OAS1* were examined using qPCR. Statistical analysis was performed on data from independent experiments (n ≥ 3), with comparisons to the poly(I:C)-activated Vector group using one-way ANOVA followed by multiple comparison correction (**P* < 0.05, ***P* < 0.01, ****P* < 0.001, and *****P* < 0.0001).


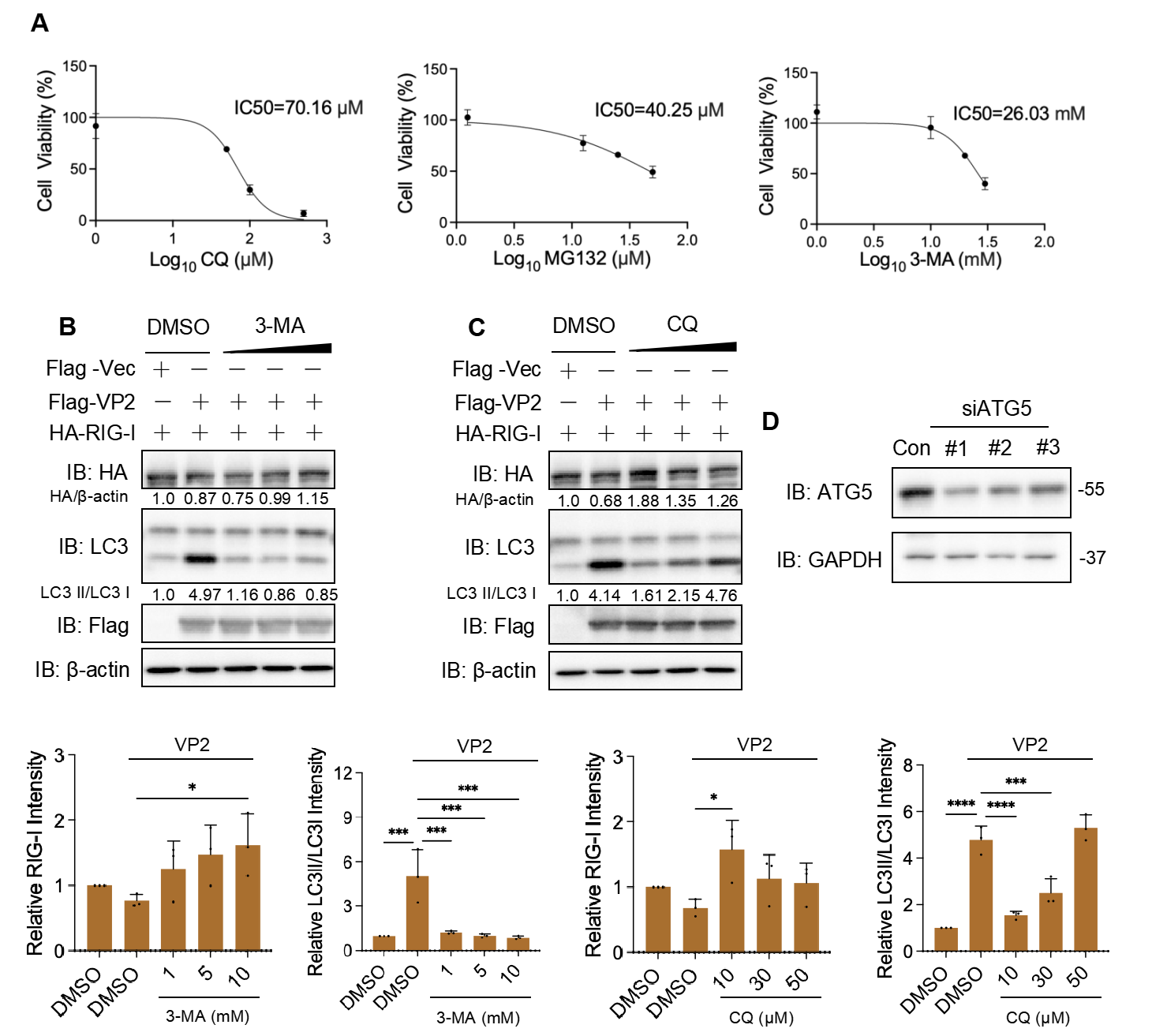


**Fig. S4 Blocking autophagy could attenuate the reduction of RIG-I induced by VP2.** (A) HEK293T cells were treated by CQ, MG132 and 3-MA in different concentrations for 12 hrs. The IC50 were calculated by CCK8 assay. (B, C) HEK293T cells were co-transfected with VP2 and RIG-I plasmids. At 24 hpt, cells were treated with the 3-MA (1, 5, 10 mM, B) or CQ (10, 30, 50 μM, C) for 12 hrs. The cells were analyzed by immunoblotting. Gray-scale statistical analysis of RIG-I relative to β-actin and LC3II relative to LC3I are displayed below. (D) HEK293T cells were transfected with siCon and siATG5 and cultured for 24 hrs, and cells wes analyzed by immunoblotting. Data from independent experiments (n ≥ 3) were statistically analyzed using one-way ANOVA with multiple comparison correction (**P* < 0.05, ***P* < 0.01, ****P* < 0.001 and *****P* < 0.0001).
